# Participatory Design of an Online Self-Management Tool for Users With Spinal Cord Injury: Qualitative Study

**DOI:** 10.2196/rehab.8158

**Published:** 2018-03-21

**Authors:** Sonya Allin, John Shepherd, Jennifer Tomasone, Sarah Munce, Gary Linassi, Saima Noreen Hossain, Susan Jaglal

**Affiliations:** ^1^ Department of Physical Therapy University of Toronto Toronto, ON Canada; ^2^ School of Kinesiology and Health Studies Queen's University Kingston, ON Canada; ^3^ College of Medicine University of Saskatchewan Saskatoon, SK Canada

**Keywords:** health education, internet, spinal cord injuries, self-management

## Abstract

**Background:**

Rehospitalization rates resulting from secondary conditions in persons with spinal cord injuries (SCI) are high. Self-management programs for many chronic conditions have been associated with decreases in hospital readmissions. However, in the SCI community, evidence suggests that satisfaction with traditional self-management programs is low. Users with SCI have indicated preference for programs that are online (rather than in-person), that target SCI-specific concerns, and are led by peers with SCI. There is currently no program with all of these features, which addresses self-management of secondary conditions after SCI.

**Objective:**

The aim of this study was to provide details of a participatory design (PD) process for an internet-mediated self-management program for users with SCI (called SCI & U) and illustrate how it has been used to define design constraints and solutions.

**Methods:**

Users were involved in development as codesigners, codevelopers, and key informants. Codesigners and codevelopers were recruited from consumer advocacy groups and worked with a core development team. Key informants were recruited from geographically distributed advocacy groups to form a product advisory council that met regularly with the core team. During meetings, codesigners and informants walked through stages of work that typify PD processes such as exploration, discovery, and prototyping. This paper details the process by analyzing 10 meetings that took place between August 2015 and May 2016. Meetings were recorded, transcribed, and subjected to an inductive thematic analysis; resulting themes were organized according to their relationship to PD stages.

**Results:**

A total of 16 individuals participated in meeting discussions, including 7 researchers and 9 persons with SCI from 4 Canadian provinces. Themes of trust, expertise, and community emerged in every group discussion. The exploration stage revealed interest in online self-management resources coupled with concerns about information credibility. In general, participants indicated that they felt more confident with information received from trusted, in-person sources (eg, peers or health care professionals) than information found online. The discovery stage saw participants propose and discuss concepts to filter credible information and highlight community expertise, namely (1) a community-curated resource database, (2) online information navigators, and (3) group chats with peers. Several tools and techniques were collectively prototyped in an effort to foster trust and community; these are illustrated in the Results section.

**Conclusions:**

A PD process engaging users as codesigners, codevelopers, and informants can be used to identify design concerns and prototype online solutions to promote self-management after SCI. Future work will assess the usability of the collectively designed tools among a broad population of Canadians with SCI and the tools’ impact on self-efficacy and health.

## Introduction

### Self-Management of Spinal Cord Injury

Managing a spinal cord injury (SCI) is a lifelong process. Within the first year of injury, more than half of the people discharged with SCI may require rehospitalization due to a secondary condition (eg, a pressure sore); even 20 years post injury, rehospitalization rates remain over 30% [1]. Rehospitalization rates in Canada have remained high for more than 10 years [2], whereas, at the same time, length of stay in inpatient rehabilitation has decreased dramatically [3]. Therefore, there is a growing need to emphasize health management support for persons with SCI in the community. Self-management is one support option; this has been described by Barlow et al as an “individual’s ability to manage the symptoms, treatment, physical, and psychosocial consequences and lifestyle changes inherent in living with a chronic condition” [4]. Effective self-management, Barlow and colleagues explain, requires the ability to “monitor one’s condition and to affect the cognitive, behavioral, and emotional responses necessary to maintain a satisfactory quality of life” [4]. In the SCI community, poor self-management has been identified as a factor in the development of an inactive lifestyle and secondary conditions [5,6].

Self-management programs encourage self-management through activities such as symptom monitoring, medication management, problem solving, and health-related decision making [7]. Established community-based programs, such as Stanford’s chronic disease self-management program (CDSMP) [8] and the UK’s expert patient program [9], rely on trained peers to guide activities for groups comprising people with different chronic conditions [8,9]. Both programs have been associated with positive health outcomes such as improvements in health-related self-efficacy [7-9], lower hospitalization rates [7], and reduced health care expenditures [10,11]. However, evidence suggests that they do not effectively address the needs of persons with SCI. For example, a qualitative study on the experiences of CDSMP participants with neurological conditions (eg, stroke, multiple sclerosis, and SCI) saw participants with SCI reporting the least program satisfaction [12]. Participants with SCI and group leaders both suggested that SCI-focused groups (eg, groups with modules adapted for SCI-specific concerns) would be preferable to the SCI community [10]. These findings were underscored by a Canadian survey where the participants with SCI expressed a desire for condition-specific self-management programming, mentoring by peers with SCI, and virtual or online participation [13].

### Virtual Self-Management Support

In response to the need for targeted and remote programs, telephone-based programs have emerged [14-17]. Participants in these programs have reported high ratings for their experience [14,15], left with improved levels of activation, social participation, and awareness [16], and have found information presented to be credible [17]. However, participants in the Canadian survey indicated a distinct preference for online-program delivery over phone delivery [13]. In addition, participants of telephone-based programs have reported difficulty in assembling program information [15]; an online program may mitigate this problem by centralizing information.

Consistent with these data is the evidence showing that the SCI community increasingly turns to the internet for self-management information. In a 2008 survey of almost 3000 US residents with SCI, approximately 65% reported using the internet and most claimed to be online daily [18]. Similar results were produced by a 2014 survey of 500 veterans with SCI [19]. For those with internet access, educational videos addressing management of SCI secondary conditions (eg, managing or preventing pressure ulcers or pain) have increased health-management knowledge and encouraged behavior changes (eg, the adoption of hypnosis) [20,21]. E-learning modules on topics such as pressure ulcer or bladder management have been linked to increased management knowledge [22-24] and internet usage has generally been associated with emotional health [25,26]. However, despite the benefits of internet for the SCI community, there is still no known tailored and internet-based self-management program.

### Design of an Online Program

In 2012, to help fill the need for high-quality online self-management support, a team of researchers including a lead author (JS) created several short e-courses for people with SCI [22,23]. This paper documents efforts to extend this online service to include peer-led self-management support. The name of the extension, SCI & U, is a gesture to the project’s relationship to SCI-U and a reference to the peer connections that form the basis of successful self-management programs, such as My Care My Call [15,16] and SCI Action Canada [14,17]. SCI & U was initiated by stakeholders funded by the Rick Hansen Institute to explore self-management; these included rehabilitation researchers, users with SCI, and clinicians [27].

To increase the likelihood of users accepting the resulting self-management tools, a participatory design (PD) approach was utilized, which includes people with SCI as codesigners and informants. This paper describes the process in detail and illustrates how it has been used to define design constraints and create solutions that have been prototyped [28].

## Methods

### The Participatory Design Process

Knowledge about how to manage the health-related consequences of SCI may be possessed by persons with SCI implicitly rather than explicitly, that is, it may be tacit. For example, persons with SCI have expressed difficulty articulating sensations during wheelchair selection [29]; however, this input is critically relevant to accessing appropriate care. PD is an iterative design and research process that acknowledges the importance of tacit end user knowledge and attempts to access it by involving users. A central PD concept is that participatory action expresses tacit knowledge and encourages sensitivity. Although the mechanisms for user involvement vary from project to project, the primary goal of PD is to improve users’ quality of life [30]. Typical PD processes see users involved in design continually and in a sustained fashion [31] either as informants (eg, via focus groups and key informant interviews) or as codesign partners (eg, partnered with design and development teams) [32,33].

When related to lifestyle promotion applications, PD processes have been found to empower and educate users as well as encourage application adoption and effectiveness [33]. Such benefits may have special relevance to persons with SCI, as people with SCI (and groups of disabled users, more generally) are often not consulted during the design of the health interventions that target them [34]. This is despite the recognized utility of community consults by national consumer advocacy organizations [35].

**Figure 1:**
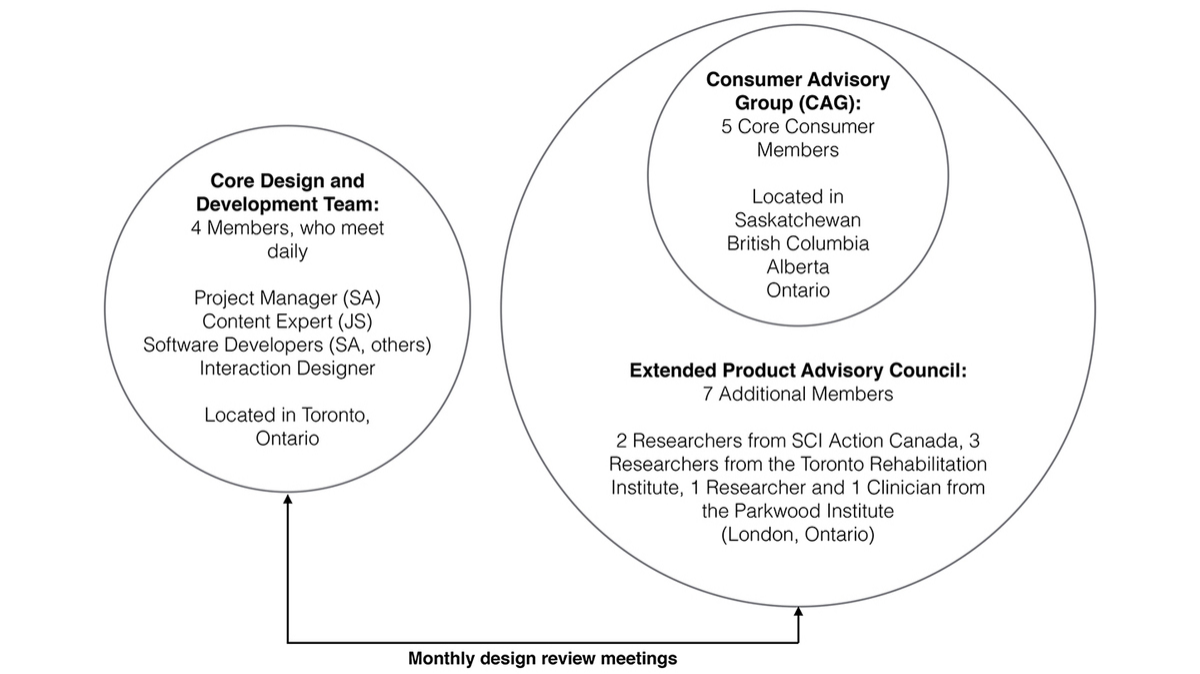
The core design and development team met daily; designs were refined at monthly meetings with a Product Advisory Council containing 5 core users with SCI (the CAG). SCI: spinal cord injury.

Figure 1 shows that a 4-member codesign and codevelopment team met daily to create product designs and prototypes; designs were refined at monthly meetings of a product advisory council. This council contained 5 core users with SCI (the “CAG”).

To maximize potential benefits of PD during the development of SCI & U, potential end users have been embedded as both program informants and codesigners. The organizational structures used to facilitate this involvement are illustrated in Figure 1 and described as follows:

#### Codesign and Codevelopment Structures

The core design and development team consisted of 4 individuals and was co-led by 2 researchers (JS and SA). One (JS) was a person with SCI, who was closely involved with the development of online health information resources for the SCI community [22,23] and the other (SA) was a human-computer interaction researcher. Additional design and development members were recruited through SCI-Ontario, an Ontario-based consumer advocacy group. These additional members, who were people with SCI, included the project’s lead programmer and an interaction designer. The core design and development team interacted regularly and met weekly.

#### Informant Structures

The codesign team was informed by monthly interactions with a product advisory council. Original members of this council were 5 geographically distributed people with SCI (called the Consumer Advisory Group or CAG) who were recruited through Canadian SCI advocacy organizations (eg, SCI-Ontario, SCI-British Columbia, and the Rick Hansen Institute). Recruitment was designed to promote diversity; original members were from several Canadian provinces (Saskatchewan, British Columbia, Ontario, Alberta), from both rural and urban areas, and reflected lived experiences with different levels of injury. The size of CAG was designed to capture differing perspectives while allowing everyone’s meaningful participation in discussions (groups ranging in size from 8-12 are typically recommended in qualitative research [36]). Membership also rotated annually. Other stakeholders from clinical and research communities were also invited to participate in periodic discussions; these individuals included physical activity experts from SCI action Canada and a dietitian from Parkwood Hospital in London, Ontario.

Activities undertaken during group meetings have, to date, loosely followed the stages of PD described by Spinuzzi [31], which are as follows:

#### Exploration

During initial meetings, participants described experiences with self-management and use of internet to facilitate health and well-being. To encourage discussion, members of CAG were asked to independently review 5 to 10 online resources designed to support independent self-management. Online resources were discovered based on literature reviews and internet searches. Resources with interactive features (eg, resources that provided feedback on symptoms or treatments) were prioritized and have been correlated with positive health outcomes in reviews of health-information technologies [37]. Examples of selected resources include e-learning modules (eg, [23]), discussion forums (eg, [38]), and sites with community reviews (eg, [39]).

#### Discovery

After discussion of online self-management strategies and tools, the group fleshed out features for a first iteration of novel online programming. Key concerns and barriers constraining the development were also identified during these discussions.

#### Prototyping

Finally, the group worked to prototype proposed features in such a way so as to mitigate concerns and barriers identified by the group. Concepts were translated into drawings and interactive wireframes by the core design team and were iterated upon, based on the group feedback. To date, several concepts have been built into a functioning prototype, which is currently accessible online (at http://www.sci-and-u.ca).

### Data Analysis

In the sections that follow, we analyze the content of the first 10 meetings between the CAG members of the product advisory team and the core development team. Meetings took place between August 2015 and May 2016; each meeting lasted about 90 min and was mediated via Skype and digitally recorded in the MP4 format using Call Recorder (eCamm Network, Sommerville, Massachusetts, USA). Resulting MP4 data was professionally transcribed and the accuracy of transcripts was verified by the lead author (SA).

Transcripts were analyzed using an inductive thematic analysis using Nvivo 10 software (QSR International, Doncaster, Australia). The paradigm that guided this analysis was pragmatic and focused on discussion around specific phenomena, that is, the use of internet to support self-management activities. It has been argued that a focus on specific phenomena is well suited to health-services research as it caters to both qualitative and quantitative analyses [40].

Codes representing key themes were identified in transcripts by the 2 authors (SA and SH) as per the instructions of Braun and Clark [41] and organized around PD stages outlined by Spinuzzi (eg, exploration, discovery, and prototyping) [31]. Meetings with additional authors (JS and SM) were held to discuss and resolve discrepancies in coding and to decide the umbrella “labels” for resulting themes. It is to be noted that one of these authors was a summer student in health systems (SH) and the other (SM), a knowledge-translation researcher with experience in qualitative analysis. Changes to the coding scheme were made iteratively and by consensus between 3 authors (SA,SM, and JS). Once the consensus was achieved, the lead author organized codes so as to highlight core concepts.

This process received Research Ethics Board approval from the University of Toronto (REB # 26429), and all individuals who participated in meetings consented to participate.

## Results

A total of 16 people participated in the 10 meetings between the product advisory council and the development team. These included 8 individuals with SCI (3 on the core design and development team, 4 in the original CAG, and 1 on the extended product advisory council) and 1 person with cerebral palsy (who was an original member of the CAG). Additional members of the product advisory council included 2 members of the SCI Action Canada research team and rehabilitation researchers from the University Health Network/University of Toronto and the Parkwood Institute in London, Ontario. Twelve meeting participants were from Southern Ontario (Toronto, London, Kingston, or Waterloo); remaining participants came from Saskatchewan, British Colombia, and Alberta. Among the 8 participants with SCI, 5 were from Ontario, 3 were women, 3 had injuries above the T1 level, and all had been living independently with SCI in the community for more than 5 years. Although the CAG and core design and development team were present at most meetings, other attendees were present only at 1 or 2 meetings when topics of relevance to their expertise were discussed.

**Figure 2:**
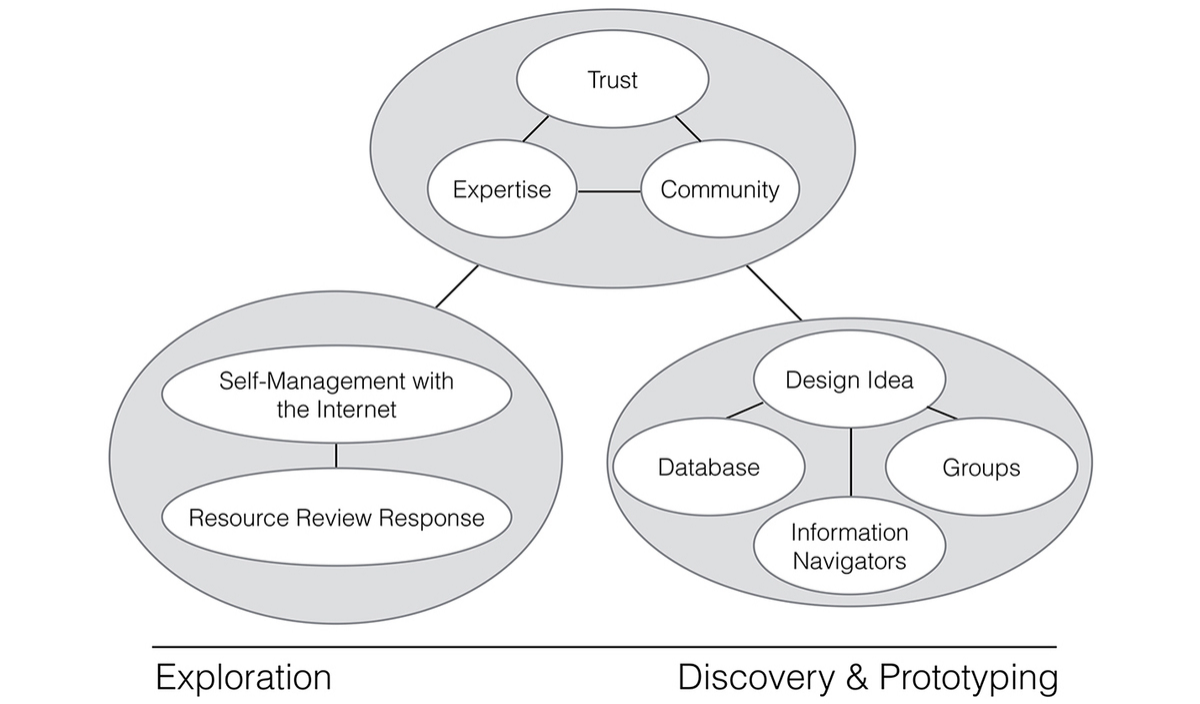
A thematic map illustrating key concepts and their relationship to the PD stages of Exploration, Discovery and Prototyping.

A thematic map illustrating high-level themes can be found in Figure 2. Themes of trust, expertise, and community were represented in every group conversation, whereas other themes were focused around particular PD process stages. For example, themes labeled “Self-Management” and “Internet and Resource Review Response” were largely confined to transcripts of Exploration Stage meetings. In contrast, themes associated with idea generation were more commonly found in later transcripts, during Discovery and Prototyping stages.

In the results that followed, we teased apart high-level themes and illustrated them with representative quotes. Quotes are identified by number rather than name to protect the participants’ anonymity.

### Stage One: Exploration

During initial meetings, participants described a wide range of techniques and services they had used to support and maintain their health when living in the community. These included the services of community-based organizations (eg, ParaSport New Brunswick), health care practitioners (personal trainers or physical therapists), and peer-support networks.

#### Self-Management and Internet

Internet, however, was described as playing an important role in self-management for participants with SCI, as it had helped them to do the following:

##### Discover Services or Interventions

Internet forums helped 1 participant decide whether or not to get a colostomy; as he was making this decision, he explained that:

looking at forums...helped me get my head wrapped around a few things and come out thinking more clearly.

ID #1

Others acknowledged online-discussion groups and forums to be sources of community as well as information. According to 1 CAG member:

...interacting with other people with the same kind of problems is probably a useful thing...if for no other reason than to know you’re not alone.

ID #2

Some participants described using internet to locate specific community services. For example, one member of CAG described taking charge of and modifying her personal environment with the help of a contractor she located on the internet; she had communicated with this person using images from her phone.

It was very useful because there were lots of things that I couldn’t explain verbally...but once he saw a picture it’s like...oh, okay, I get it.

ID #3

##### Prepare for Meetings With Health Care Professionals

Participants described performing internet searches to prepare for meetings with health care practitioners. As one participant explained:

I look to get enough information [from the Web] so that I sound educated when I go speak to a professional, whether it be my personal trainer or my physician or my OT for seating and wheelchairs. I want to be informed before I go and advocate for what it is I think I need.

ID #5

Internet searches were also described as being useful to determine whether HCP visits were, in fact, required. This was explained by one participant in this way:

I like...to piece together all the puzzle pieces and to go, ‘yeah, okay, that is the thing I want to do’ or ‘no I don’t need to go to the doctor’ or ‘yes, I do need to call Emergency.’

ID #3

##### Revisit Skills Learned in Rehab

Many participants described the period of time surrounding discharge from rehabilitation as particularly overwhelming; although self-management information was provided during inpatient rehabilitation, not all patients were ready to absorb it all. As one participant stated:

...when we’re in rehab, we get more information than we would admit to getting but we just don’t process it. And so, shortly after injury, when you’re back at home...then the things you didn’t pay enough attention to in rehab become salient as a problem.

ID #6

It is at this point that participants remembered searching for information on the Web. As one participant explained:

...that’s when you may be looking for how-to [videos] related to self-management concerns.

ID #3

##### Access Research Information

People more distant from inpatient rehabilitation, however, were described as having different information needs and as more likely to use the internet to research particular interventions or services in depth. One participant said:

It depends on where you’re at in your post-injury life. I’m more keen to look at research-based content than...step-by-steps.

ID #1

Another participant defined self-management shortly after injury as being about “managing an unknown entity” while now, several years later:

...it is [about] tertiary conditions and how my disability interacts with those.

ID #2

This same participant described using the internet to locate research reports and inform self-management decisions. One had influenced him to stop taking fiber pills:

...when I read that report, I thought...well I’ll just see what happens. I was kind of surprised at the results...and I shared that with my family doctor.

ID #2

##### Ease the Burden of Travel

While several participants explained that they had taken advantage of peer-support services after their injury, one participant indicated he had used Skype to deliver peer support to a colleague:

I establish a Skype contact with her…it was interesting to establish the contact and then figure out what needs she has and what I can do to help. It’s very useful for both of us.

ID #1

Others similarly described use of videoconferencing tools to access social support or employment. One participant explained he had once attended group meetings in person, requiring him to drive more than thirty minutes and endure pain as a result. Videoconferencing improved his situation. He stated:

I’m in a lot of pain. I’m just about to head out the door and I think, someone suggested you can use Skype. I think it is an effective way to meet, for sure.

ID #2

#### Resource Review Responses

Independent review of online resources for self-management also generated conversation around several themes, including:

##### Lack of Familiarity

Although participants reported using the internet to support and maintain their health, many were unfamiliar with online resources presented for review. For example, when reviewing forums for users with SCI to exchange health information, one participant commented that he had:

...never used such a forum...didn’t know they even existed.

ID #1

Other participants indicated that, although they may have used forums to decide on things to buy, they had never considered using forums for self-management decisions. At the same time, participants responded positively to online resources they were asked to review. One participant, who was not only a person with SCI but also a clinician, indicated she would be sharing details about discussion sites for accessibility products with “patients … looking at home modifications for discharge” [ID #8] based on the group discussion. Forums containing personal stories of treatment or recovery were also found to be useful and appealing; 1 participant felt they allayed “fears and … trepidation” related to care decisions [ID #2]. Sites containing community ratings or discussions of care provided by local clinicians were similarly unfamiliar, yet described as “really interesting” [ID #5] or “quite unique” [ID #8] by participants in the group.

##### Appreciation of Diversity

In addition, most participants indicated they were impressed by the range of self-management resources available online and felt this diversity had utility. One participant explained that she “liked a lot of links” [ID #3] to follow when doing online research related to care, while another acknowledged:

...everybody is different and different people are going to find different things useful. It’s a tricky thing to know who is going to want what or trying to second guess what people would be interested in.

ID #2

##### Questions of Trust

Despite the appeal of the online resources, participants with SCI expressed strong concerns related to the credibility of online information. The group clearly preferred self-management information obtained in-person from trusted sources. As one participant explained:

I’m more likely to take the advice of a trusted friend, doctor or service coordinator...rather than going to the internet.

ID #2

Even users of internet forums described online information as less credible than in-person information:

It’s a lot of crap in those forums.

ID #2

I’m always a little skeptical of Joe Public.

ID #4

Moreover, participants characterized users of online self-management tools as potentially vulnerable to bad information or advice.

People might be overwhelmed if they’re looking for this information.

ID #7

If you’re in a lot of pain, you’ll look at whatever.

ID #2

Protecting vulnerable or compromised users from misinformation was understood to be important:

Some people really need this information and we need to do our best to implement safety measures for them.

ID #7

At the same time, limiting exposure to information, or acting as an information “censor” was described by one participant [ID #1] as contrary to users’ need for information diversity.

What defined credibility of online information varied from participant to participant. Some expressed feeling confident when information was clearly associated with reputable health care institutions, that is, when “coming from health care professionals or something” [ID #2]. Others perceived information from health care professionals and research institutions to be incomplete or biased; research information was described by one participant as “based on such narrow criteria...when there’s so much more” [ID #4]. In addition, participants prioritized information associated with peers as it was based on “personal experience,” and therefore more likely to address “what works with us” [ID #9]. In general, participants expressed a need to be clear as to the meaning and labeling of expert or credible self-management information; as was stated:

We need to come up with some very specific ideas [as to] how we’re defining those terms.

ID #7

##### Questions of Accessibility

The discussion of existing online self-management resources raised accessibility concerns related to the use of technology and health care services. Persons with SCI experience significant care limitations; it was felt that internet resources might not be sensitive to these. Sites designed for communities to share information about clinicians, for example, were questioned because users of clinical services for SCI often “don’t have choice” [ID #3]. As one participant noted, if he were to discover that a clinician profiled on a website had a “bad rating, well, then there isn’t a hell of a lot I can do about that” [ID #2]. Another participant who was both a clinician and a person with SCI expressed concern that finding doctors or services to be “rated poorly” might decrease users’ “confidence” in care [ID #8], thereby taking away from self-management efforts.

Diversity of physical accessibility issues were also revealed by the resource review. For example, while one participant responded positively to short videos of self-management strategies claiming that these catered to her “YouTube mentality” [ID #3], another found the same videos challenging to operate. As this second participant explained:

I’d like to read [information] so that I can re-read it, rather than trying to get my...hand on the [video control] to scrub it back 5 seconds.

ID #2

Interest in and ability to access information on mobile devices was similarly varied. Although some participants responded positively to the idea of mobile-friendly services, one participant with a high-level injury indicated he could “not really use a (mobile) phone” or tablet and had found tools to promote accessibility of these devices, such as voice activation, to be “anything but relaxing” to configure [ID #2]. Participants with comparable injuries, however, reported different experiences with the same devices. One explained he used “commercial products” such as the Tecla Shield (Komodo OpenLab, Toronto, Ontario) [42], which facilitates access to touchscreen devices via assistive buttons and other controls [ID #6]. This had allowed him to comfortably use a mobile phone despite having high-level injury, and his mobile phone was his primary device.

In summary, discussions during the exploration phase demonstrated value in exposing users with SCI to a wide variety of interactive self-management resources. At the same time, conversations highlighted the need to organize and promote information credibility and to accommodate individuals with very different accessibility needs.

### Stage Two: Discovery

To mitigate credibility concerns, the discovery phase focused on mechanisms to help filter, or lend credibility to, online self-management information. The mechanisms proposed were as follows.

#### A Community-Curated Resource Database

In response to the diversity and quantity of online self-management resources, participants proposed the creation of a collaborative database for self-management information that might operate something like a Wiki. As was explained:

Not all [Wikis] have immediately useful information but they are a very good start for me to add data.

ID#1

Others responded positively to this idea, suggesting such a tool might help filter through information and be better than:

just going on Google [for information about] a new chair or an accessible vehicle or whatever.

ID #5

Several ideas for information vetting (eg, using moderators) were floated to allow high quality information to be more visible or accessible.

#### Online Information Navigators

Discussions made it clear that information from in-person resources was perceived as more trustworthy than information from online collectives. In response, participants proposed the idea of internet-accessible information navigators. As one of the CAG members explained:

...rather than let 1000 people express their opinion...it’d better to go to an expert; [online] experts...might filter out useless [database] contributions.

ID #1

Others similarly agreed that “a direct resource that you can communicate with” would be preferable to a database with comments or reviews written by “a bunch of guys who know as much or less than you” [ID #7]. A use-case scenario was detailed in which a user might interact with an online peer using a webcam. The online peer, she explained, might suggest resources based on this information, that is:

...say...you could use this, you could use that, here are a couple of links for the equipment for grips or whatever.

ID #5

Others received this idea positively, agreeing that:

...it’s good to have somebody to help you [learn] what...to look for.

ID #1

#### Online Groups

For some participants, the act of meeting regularly with peers and researchers during the exploration phase increased awareness of self-management strategies and techniques. For example, one participant explained that he had experimented with different management strategies for bowel care based on resources he encountered during the exploration phase and found that videoconferencing sessions with CAG helped him “build a relationship” around the experience [ID #2]. Another participant, who lived in a relatively remote part of Canada, similarly described the value she found in sharing information about self-management as a result of the PD sessions. As she explained:

I like these meetings. I know people with SCI but the community is relatively small. The ability to connect with...people that “get it” because they’re in the same situation...that’s what I’m most excited about.

ID #5

### Stage Three: Prototyping

On the basis of this input, the group began fleshing out design concepts for a resource database. During prototyping, the core design and development team worked to accommodate diverse interests and perspectives; this meant, for example, that information in both video and text format was highlighted and a responsive framework (ie, one that could conform to phone, tablet, and desktop displays) assumed. Prototyping involved creating drawings and interactive wireframes. Wireframes were subsequently translated into a functioning prototype located online (as of June 1, 2017) [28]. An open-source platform called Ruby on Rails (version 3.2) was selected for development of functional prototypes so as to enable end users to contribute.

**Figure 3:**
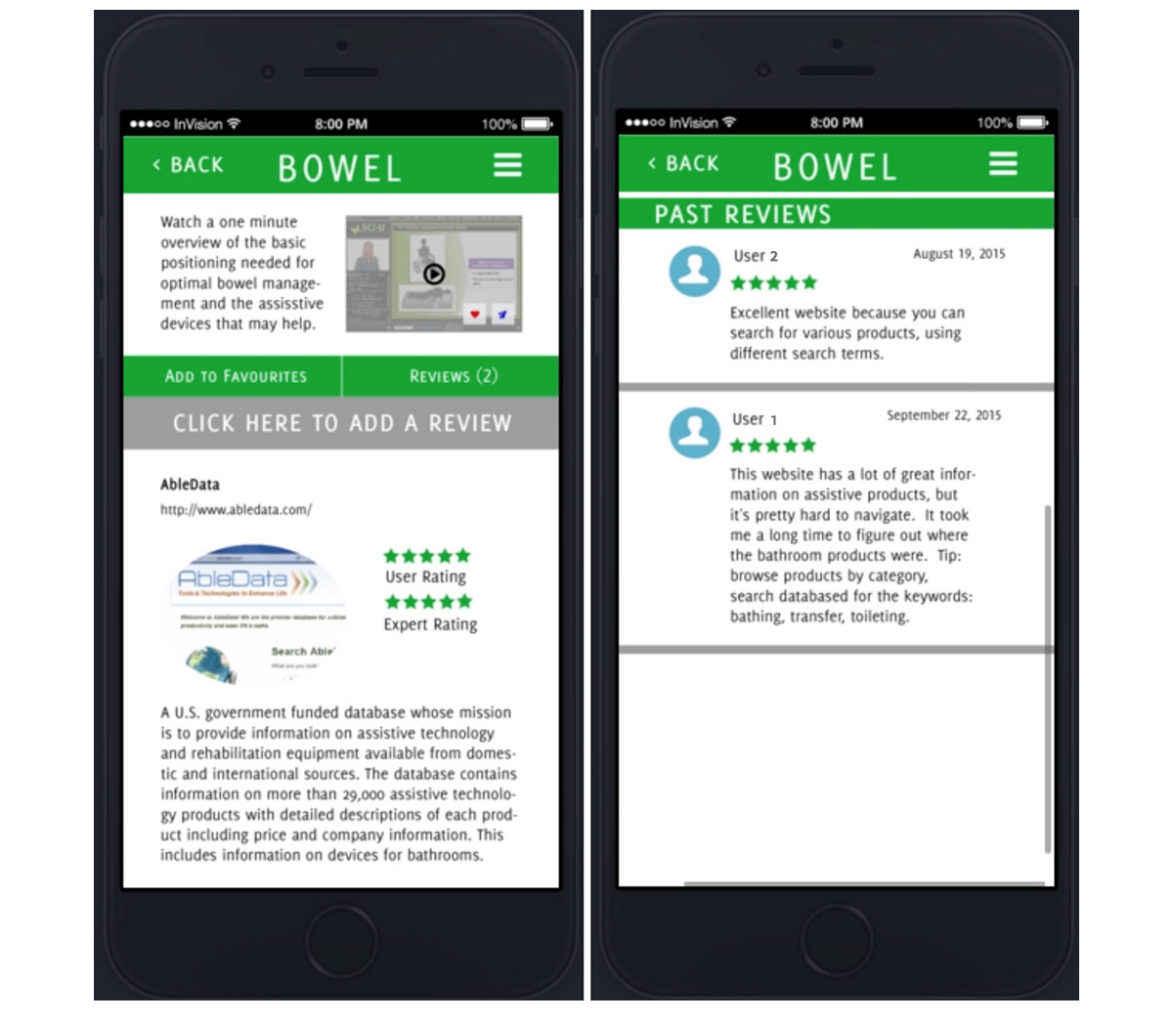
Information Wiki or resource database entry, with star ratings from experts and others.

**Figure 4:**
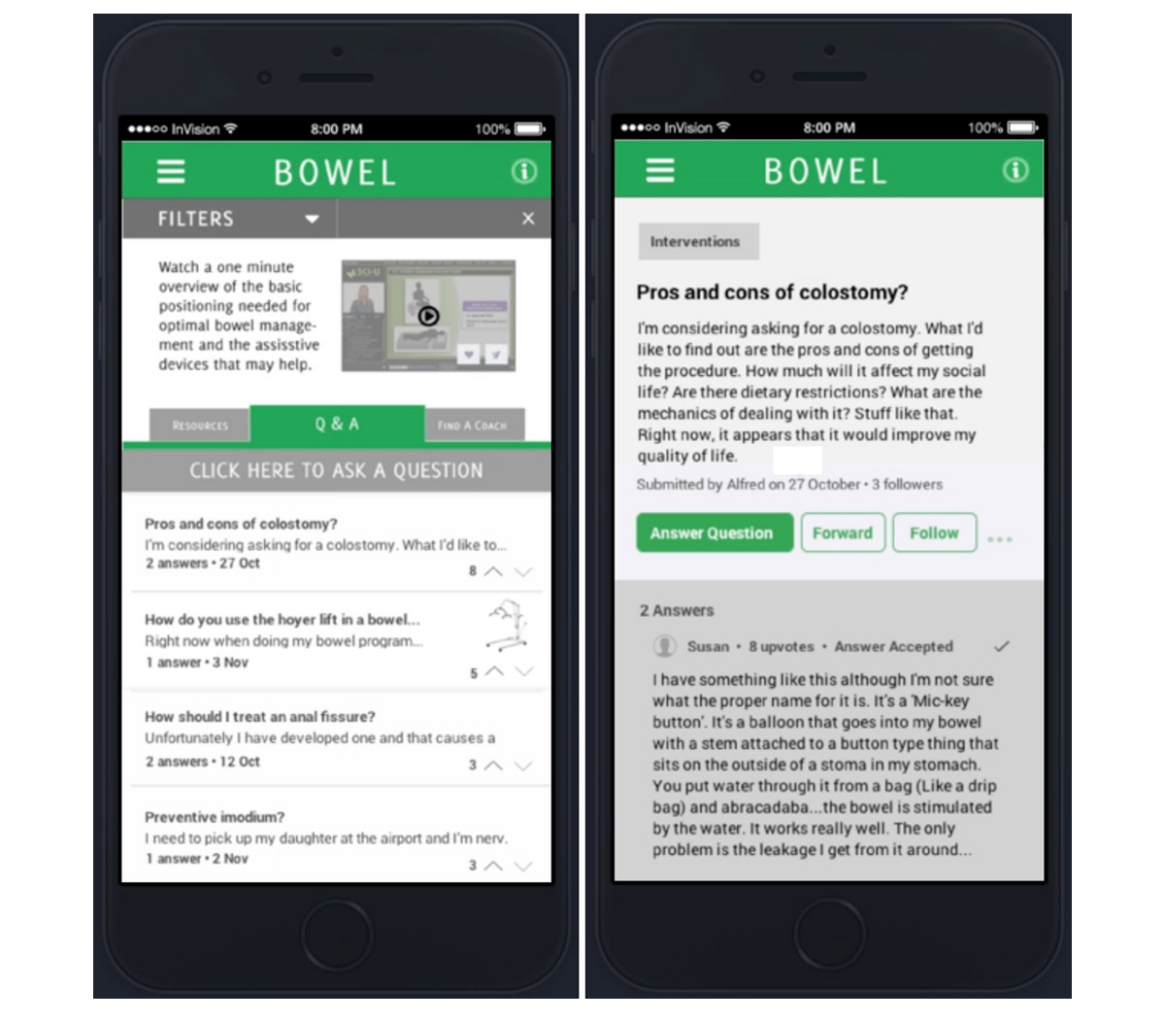
Example of up-voting online information.

To illustrate the benefit of the PD process, we describe prototyping tools and techniques to identify credible content. The tools, and the discussion surrounding them, are described in Figures 3 and 4.

#### Stars and Up-Voting

The core team proposed enabling community ratings (ie, stars) to label credible information in the database. An illustration of a star-based rating system as it was presented to CAG is shown in Figure 3.

Participants responded positively to this idea but were concerned that ratings might lose value when aggregated over a large community of users. As one group member explained:

...absolutely there’s value in ratings from the user population, but the challenge with anything online and open...is that some [ratings] will be valuable and some will not.

ID #4

In response, the core team proposed ratings from an expert panel that were distinguished from those of the broader SCI community. Participants also created a formula for the ideal membership of this panel. Consensus was that it should be diverse and include “health care practitioners, researchers, and people who are living (with SCI) every day” [ID #3]. As one participant explained, the ideal expert panel would be one containing:

...someone who is keen but green, someone who has been around the block a few times, someone who is a frontline service provider, and someone who has got their roots in academia.

ID #4

In addition, the core team suggested enabling up-voting and down-voting of reviews to identify information with value. Up-voting of comments is a technique used by several review-based sites. On Amazon, for example, users are asked whether reviews of products are helpful, and reviews are ranked (or up-voted) based on their overall helpfulness to the user community. The concept, as it relates to designs for SCI & U, is illustrated in Figure 4.

Up-voting for SCI & U was met with similarly positive feedback; participants called up-votes “a good tool to push expertise to the top” [ID #1]. A perceived additional advantage was the idea that up-votes might be associated with, or help identify, users with a track record of high-quality reviews or responses. As one participant explained, up-voting creates “a chance for people to be able to see” who is producing high-quality information and “for [online] ‘experts’ to gain recognition for their expertise” [ID #6].

#### Expertise Points and Activity Feeds

**Figure 5:**
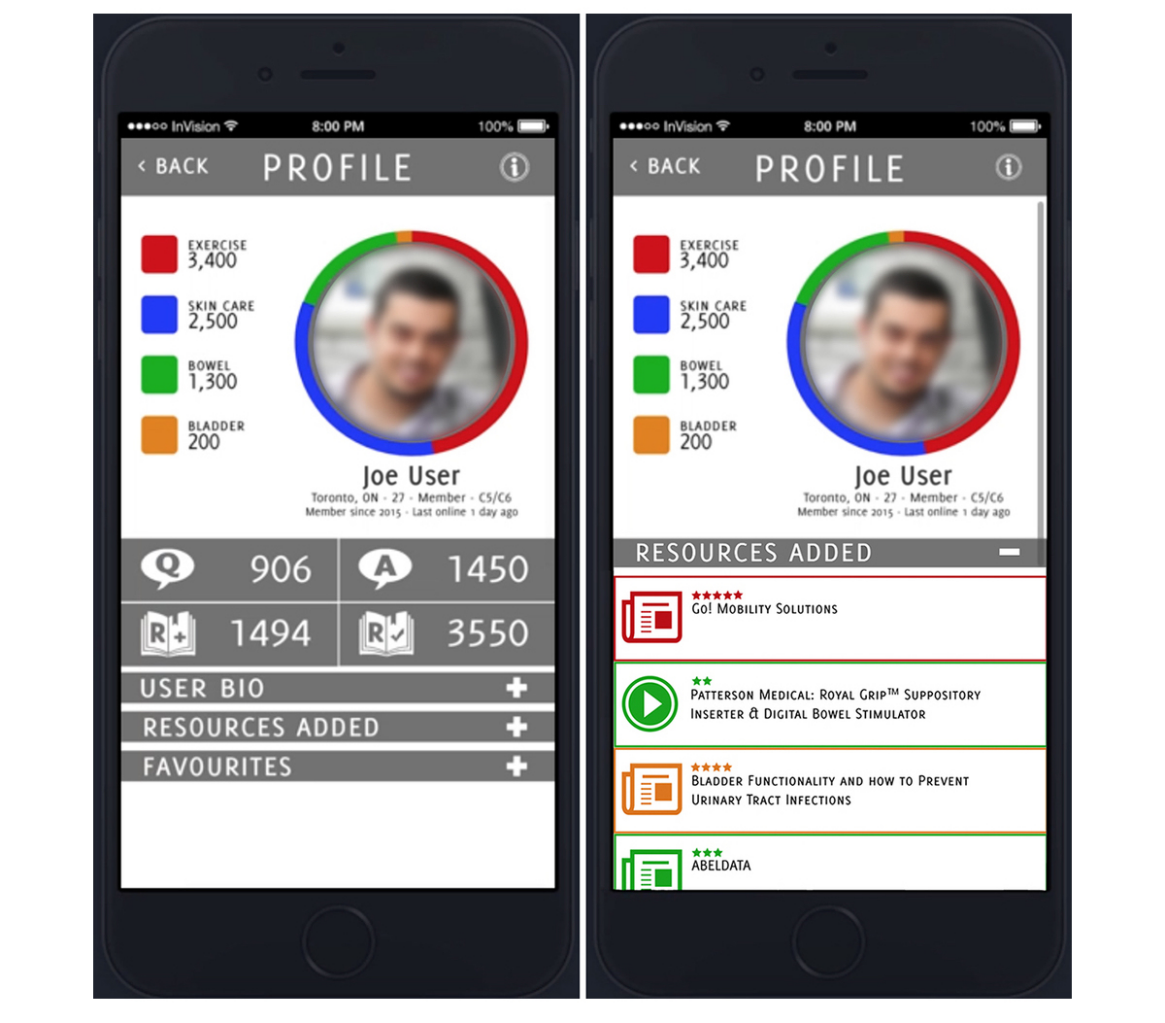
User points at left; user activity feed at right.

Up-voting led the group to consider ways one might associate expertise with content and individual users. More specifically, participants discussed the idea of rewarding contributors with “points” reflecting the value of their contributions. It was further suggested that users might gain points in specific health management areas, for example, they might gain expertise in bowel or skin management based on their contributions of information to these sections. This concept is illustrated in Figure 5.

However, the suggestion of user points was seen as controversial. One participant felt the idea risked “introducing competition” [ID #4]. Others concurred, saying that points would garner users nothing other than “bragging rights,” that is, the ability to say “I’m better because I know more” [ID #1]. Moreover, the formula for awarding points was recognized to be complicated; simply being an active contributor was not seen as sufficient to merit a badge of expertise.

People that are really active on the site may just be looking for something to do… [activity] doesn’t necessarily equate to expertise.

ID #3

At the same time, some participants perceived that recognizing frequent contributors, or users who had produced demonstrably useful information, could have meaning when associated with user profiles of potential information navigators. Participants suggested that, for individuals who were publicly identified as information navigators, points might act as a “way … to build a resume” [ID #6].

Ultimately, the group chose to incorporate activity feeds on user profiles rather than points, as shown in Figure 5. Activity feeds were seen as a relatively nonjudgmental way to illustrate user contributions without forcing comparisons between community members. One participant explained that activity feeds:

...[let you] look at the kind of comments [users] have made, and the areas they've made them in, and determine whether or not you’re going to take their two cents.

ID #4

## Discussion

### Principal Findings

Results serve to illustrate how the CAG and core development team employed a PD approach to create functioning online self-management tools, including a resource library (Wiki), a library of accessible online peer information navigators, and infrastructure to host online community discussions or events.

Discussions that took place during the development of SCI & U reflect research results demonstrating most North Americans with SCI both use the internet and turn to it for health information [18,19]. At the same time, accuracy of online health information was a concern for participants; this is consistent with research showing online health information to be variable in quality [43,44]. However, the online health information landscape is quickly changing; more recent studies of forum discussions of care for HIV, for example, have found information posted by online users to be of “good quality” when evaluated by medical professionals [45]. Trustworthy sources of information about SCI are, moreover, increasingly available online (eg, [20-22,46]).

Discussions in this study also echo findings regarding preferred modes of health information delivery in the SCI community. A 2010 review of physical activity information for SCI demonstrated a clear preference for face-to-face information delivery and for family, peers with SCI, and health professionals as information sources [47]. A preference for health information directly obtained from health professionals over information on the internet was also apparent in results from a 2016 survey of US veterans [19]. Similarly, participants in this research made it clear they trust direct communications with individuals over communications with online groups. Information received from trusted peers was especially emphasized as valuable and relevant. This prioritization of peers as information sources is echoed in results from 2011 focus groups on the topic of exercise and SCI; as one participant in this prior study explained, the experience of peers “speaks volumes to someone with an injury” [47].

However, in both this study and the studies conducted before this [47], participants with SCI were relatively distant from the time of their injury (ie, injuries had happened more than 5 years prior). Time since injury is known to influence preferred modes of health information delivery; while recently injured individuals may prefer interactive, face-to-face modes of health information delivery, passive or mediated modes of information delivery (eg, via the internet) may be more appropriate at later stages of recovery [47]. In addition, in both this study and others [48], locating information while transitioning from the hospital to home was described as particularly challenging; there is an apparent need to provide tailored online information for the SCI community at this time.

In addition to shedding light on the access and use of online health information, the SCI & U process proved to engage potential end users while building capacity and promoting information awareness. Several participants indicated that they found valuable information as a result of the PD process and one participant reported modifying behavior based on information shared during meetings. Such process-related benefits are common to PD, as its focus rests primarily on the development of participants and organizations; tools are seen as subsidiary [30,31]. Moreover, PD processes are strongly aligned with the increasing emphasis on “person-centered” health care [49]. Groups that have adopted participatory methods in the design of self-management interventions for the SCI community are small in number [14-17,50], and the process described here extends these methods to the development of supporting technologies.

### Limitations

There are several limitations to results. Most notably, those involved in the participatory process were small in number; experiences or perceptions of the group therefore cannot be guaranteed to generalize to the experiences and perceptions of a broader community of users with SCI or SCI stakeholders. Nonetheless, efforts were made to ensure the CAG’s diversity with respect to geography, sex, and injury levels, and results obtained reflected findings associated with surveys of larger populations of individuals with SCI. This potential limitation of our study is, moreover, a commonly cited limitation of participatory processes, that is, extensive involvement of users may result in designs tailored to the needs of a small group [51].

In addition, participant selection was biased as all participants have regular access to high-speed internet. However, research indicates a significant proportion of the SCI community in North America (between 30% and 40%) do not have this kind of regular access, and almost 20% have never referred to the internet for health information [18]. Moreover, regular access to the internet has been found to be associated with high education level, socioeconomic status, and self-reported health status [19]. Participants with SCI in this study, then, may be individuals who are less in need of self-management support than those in the community and who are not currently online. A parallel telephone support process may prove to be better suited for more vulnerable users with limited access to online information.

### Conclusion and Future Research

In summary, a participatory process including potential users as codesigners, codevelopers, and informants has been shown to benefit the design of an online self-management resource for Canadians with SCI called SCI & U. Benefits demonstrated here include:

1. Elicitation and consideration of diverse accessibility considerations (eg, use of online video vs text, use of mobile devices vs PCs).
2. Prioritization of features and identification of core design concerns, including those related to online information credibility (eg, the need to define and highlight “quality” information).
3. Cocreation of acceptable strategies and techniques to mitigate identified concerns (eg, community ratings and reviews, access to online information navigators).

Currently, the team is working to evaluate the basic usability of the existing prototype based on input from a broader collection of end users and using the Mobile App Rating Scale [52]. This will help determine if the described process has successfully created products that serve both design participants and others in the Canadian SCI community. Once this is complete, evaluation efforts will shift to focus the tools’ impact on users’ self-efficacy (ie, confidence in the ability to independently manage their health) and health care utilization. Extension of the prototype is also taking place in order to support richer interactions with “information navigators” that are analogous to the interactions with the trained peer-health coaches of SCI Action Canada’s Get In Motion service [14,17] or My Care My Call [15,16]. A pilot trial to explore the impact of online interactions with trained peers is ongoing.

## Abbreviations

CAG: Consumer Advisory Group
CDSMP: Chronic Disease Self-Management Program
PD: participatory design
SCI: spinal cord injury
